# Usefulness of customized titanium plates for midface contouring surgery

**DOI:** 10.1016/j.ijscr.2022.107324

**Published:** 2022-06-18

**Authors:** Tadaaki Morotomi, Tomomi Iuchi, Narihiko Hirano, Mitsugu Fujita, Koji Niwa

**Affiliations:** a Department of Plastic and Reconstructive Surgery, Kindai University Faculty of Medicine, Osaka, Japan; b Plastic Surgery, Izumi City General Hospital, Osaka, Japan; c Center for Medical Education and Clinical Training, Kindai University Faculty of Medicine, Osaka, Japan; d Nagumo Clinic Osaka, Osaka, Japan

**Keywords:** Customized titanium plate, Contouring surgery, CAD/CAM, Facial reconstruction

## Abstract

**Introduction and importance:**

In our department, we have been performing bone reconstructions on a case-by-case basis using vascularized free tissue transfers and custom-made artificial bones (*HA*). While these procedures have specific advantages, they are also limited in terms of the invasiveness as well as the stability and strength of implants. In the present study, we describe the use of a *CTP* to achieve minimally invasive midface plastic surgery with the superior moldability of a *3D* structure and reliable stability compared to the use of autologous tissue.

**Case presentation:**

A total of three patients were included in the study. The patients (all female, ages: 66, 18, and 35 years) had bone malformation or hemifacial microsomia following surgery for maxillary cancer or multiple facial fractures. Based on *DICOM* data from preoperative *CT*, *3D* models were created on a computer using *CAD/CAM* techniques. The models were compared in simulations to determine the optimal structure. These *3D* models were used in additive manufacturing systems to create custom-made titanium alloy plates for facial reconstruction.

**Clinical discussion:**

Although the amount of soft tissue was insufficient in some cases, all patients were able to maintain the desired morphology without developing any complications such as infections, significant soft tissue atrophy, or implant failure.

**Conclusion:**

Our *CTP* model created by *CAD/CAM* was effective in contouring surgery of the midface as it had the superior stability and biocompatibility of titanium. Changes to the soft tissue should also be considered in order to further improve the procedure.

## Highlights

• The technical advancements have led to the development of CAD/CAM techniques.
• CTP model created by CAD/CAM was effective in contouring surgery of the midface.
• All patients were able to maintain the desired morphology.
• CTP fabricated with additive manufacturing has the advantages of titanium.

## 1 Introduction

Morphology of facial bones differ by individuals, and the bones have complex 3D structures. In particular, the midface, which includes the orbit, is an important factor that defines the impression of one's face. Thus, patients with defects in the midface often seek treatment to regain their normal social life. To date, various methods of using autologous tissues and artificial materials have been reported for the reconstruction of facial bone defects and malformations. In our department, we generally used autologous tissues, such as vascularized free flap and free bone graft, to reconstruct facial tissue defects. As suggested by Brawn et al. [1], autologous tissue transplantation is the preferred method of reconstruction in terms of the long-term outcome.

On the other hand, technical advancements have led to the development of *CAD/CAM* techniques. These techniques have enabled surgeons to create an accurate plan for reconstruction surgery and predict cosmetic outcomes [2]. In our outpatient clinic, the number of patients who request minimally invasive procedures has recently increased. Rather than autologous tissue transplants, more patients are requesting the use of implants for reconstruction. To address this trend, we have recently started the use of implants for facial reconstruction surgery. Implants that are commonly available include those that are made of HA and a customized titanium plate CTP. While these procedures have specific advantages, they are also limited in terms of the invasiveness as well as the stability and strength of implants. In the present study, we describe the use of a *CTP* to achieve minimally invasive midface plastic surgery with the superior moldability of a *3D* structure and reliable stability compared to the use of autologous tissue. We further discuss the advantages and limitations of the procedure with literature evidence.

## 2 Case presentation

A total of 3 patients were included. These surgeries were performed in all cases by the author, who has 20 years of clinical experience. This case report is compliant with the SCARE Guidelines 2020 [3].

Based on DICOM data from preoperative CT, *3D* models were created on a computer using *CAD/CAM* techniques. The models were compared in simulations to determine the optimal structure. These 3D models were used in additive manufacturing systems to create a CTP for facial reconstruction. CRANIOFIT (HOYA Technosurgical, Japan; 0.5 mm-thick), which was fabricated in additive manufacturing, was used for the *CTP*. The CTP was fit to a model created based on the mirror image of the unaffected side of the face. The morphology of the model was further adjusted for each patient based on the characteristics of the soft tissue and the morphology of the malformed bone surface. In all cases, the *CTP* was fixed directly to the facial bone using titanium screws. The result of this procedure showed that patient satisfaction had been high using CTP.

### 2.1 Case 1

**Fig. 1:**
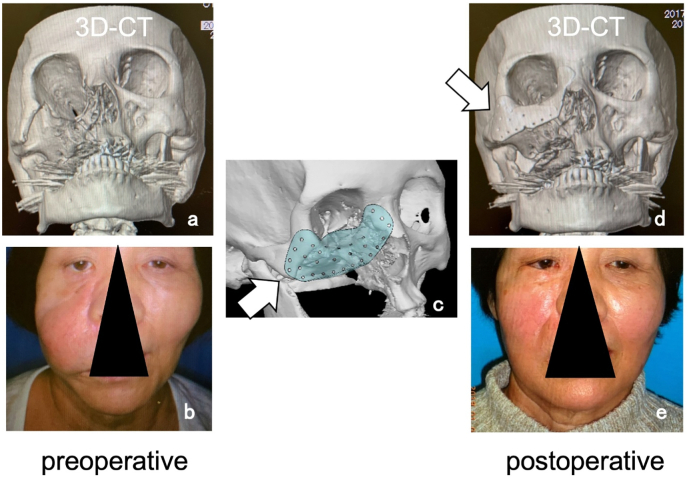
Case 1; 66-year-old, female.

The patient was a 66-year-old woman who had previously undergone surgery for maxillary cancer which resulted in right maxillary bone defect (Fig. 1-a). This patient subsequently underwent reconstruction of the right zygomatic soft tissue using a rectus abdominis myocutaneous free flap, which resulted in malformation (Fig. 1-b).

We therefore performed contouring surgery using a *CTP*. Preoperatively, we observed the maxillary defect as well as swelling of the soft tissue that was reconstructed using the flap. Based on the mirror image of the unaffected side, we created a CTP model of the buccal region including the orbital floor (Fig. 1-c, allow). The *CTP* model was designed so that it can be directly fixed over the stump of the bone defect using screws (Fig. 1-d). An incision was made to the skin to view the surgical field, and buccal reconstruction was performed using *CTP*. Excess soft tissues were trimmed to match the facial contour (Fig. 1-e).

### 2.2 Case 2

**Fig. 2:**
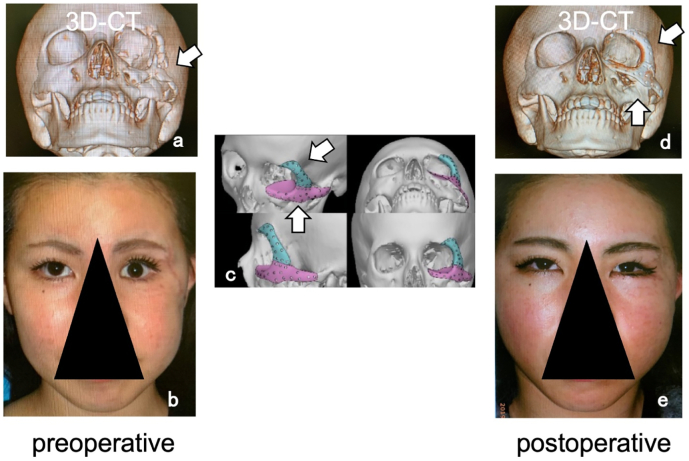
Case 2; 18-year-old woman.

The patient was an 18-year-old woman who had previously undergone open reduction and internal fixation surgery for multiple zygoma-orbital fractures (Fig. 2-a, allow). The procedure resulted in malformation of the left cheek as well as left enophthalmos (Fig. 2-b). We therefore designed a *CTP* model specifically for the purposes of contouring the left buccal region and improving the appearance of left enophthalmos (Fig. 2-c, allows). Although the left zygomatic bone was significantly flattened, there was a sufficient amount of skin and soft tissue to create the shape of the cheek similar to the unaffected side. Thus, the *CTP* model was created to reproduce the shape of the cheek, and was fixed to the orbital margin using a screw (Fig. 2-d). The surgery was successful in achieving nearly symmetrical contour and improving enophthalmos (Fig. 2-e).

### 2.3 Case 3

**Fig. 3:**
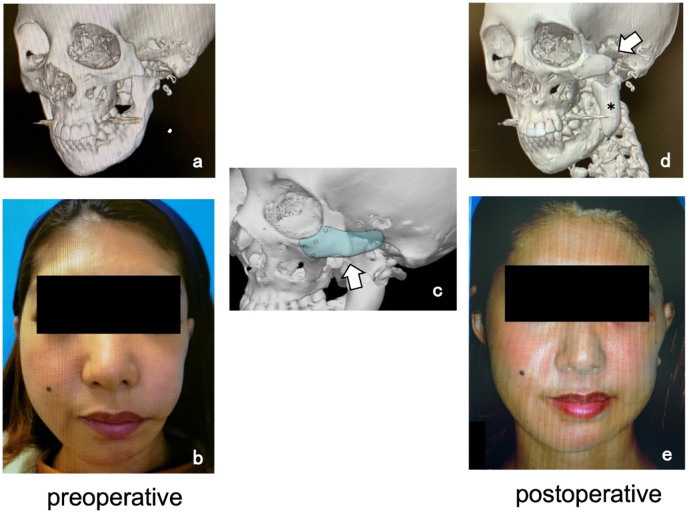
Case 3; 35-year-old woman.

The patient was a 35-year-old woman who had previously undergone otoplasty for hemifacial microsomia during childhood. When she visited our clinic, we observed malformation of the left side of her face due to hypoplasia of the left zygomatic bone and left mandible (Fig. 3-a, b). Facial contouring surgery was performed using a *CTP* to treat hypoplasia of the left zygomatic bone, and *HA* block transplant surgery was performed to augment hypoplasia of the left mandible (Fig. 2-d *). The *CTP* was fixed to the diaphysis of the zygomatic bone using screws. While the facial contour improved postoperatively, there remained some deficiency (Fig. 3-e).

## 3 Clinical discussion

Conventionally, silicone had been used in custom implants for facial reconstruction [4]. More recently, with the advancement of *CAD/CAM* techniques, implants are being made with a number of different materials and used in a wide variety of cases. Since facial bones have complex 3D structures, various methods of using autologous tissues and artificial materials have been reported for the reconstruction of facial bone defects and malformations. In our department, we have also been using *CAD/CAM* techniques in facial reconstruction procedures.

Facial reconstruction procedures based on *CAD/CAM* techniques can be classified into 4 categories based on the methods of creating artificial bones and templates [2]. According to this classification method, our technique is closest to type 1 as it is designed by CAD and fabricated by CAM in additive manufacturing. However, our procedure is unique because we use titanium in our additive manufacturing system. Our *CTP* models also allow us to design and pre-define the position of small holes for fixation. Thus, we determine the position of the small holes based on the state of the bones to which the *CTP* model will be fixed. *CTP* also has fine asperities on the surface, which helps minimize misalignments with the surrounding tissues. Thus, *CTP* differs from conventional titanium plates that are glossy and flat on the surface. In practice, the initial step of creating the model was to design the artificial bone using *CAD*, followed by some adjustments to create a *3D* image data. Based on the data, the model was created using a *3D* printer based on additive manufacturing by HOYA Technosurgical Inc.

In general, reconstruction using artificial materials is considered advantageous as it does not involve a donor and is minimally invasive. The use of artificial materials also enables the construction of complex structures and may help reduce the operative time, number of personnel involved, need for intensive care, and healthcare cost per patient as a result of early discharge [5]. A number of studies to date have reported the use of artificial materials. For example, polyethylene implants are often used in skull reconstruction [6]; however, they are not suitable for maxillofacial reconstruction as they are difficult to fix to recipients. Similarly, while *HA* is suitable for augmentation since it promotes cell adhesion and growth [7], it lacks in mechanical strength and physical stability. On the other hand, a *CTP* fabricated with additive manufacturing not only has the advantages of titanium, such as biocompatibility and mechanical strength, but also has flexibility in terms of the design and screw fixation that facilitates accurate fixation to recipients. A *CTP* is particularly considered effective for mandibular reconstruction due to its mechanical properties [8]. However, to our knowledge, there is limited evidence of its use in midface reconstruction. Despite the advantages, the use of any artificial materials including titanium is associated with the risks of damage and infection. It should also be noted that the use of artificial materials may also cause osteonecrosis and is not recommended for patients who require radiation therapy for cancer treatment [9].

In our study, none of the patients developed infection or soft tissue atrophy, or implant failure. The procedure was also successful in contouring the morphology of the midface. However, the amount of soft tissue was insufficient in some cases. Thus, in addition to considering the symmetry of hard tissue, it is also important to simulate possible changes in the morphology of the soft tissue.

## 4 Conclusion

Our *CTP* model created by *CAD/CAM* was effective in contouring surgery of the midface as it had the superior stability and biocompatibility of titanium. Changes to the soft tissue should also be considered in order to further improve the procedure.

## Abbreviations

HA: hydroxyapatite
CTP: customized titanium plate
3D: three-dimensional
DICOM: digital imaging and communications in medicine
CT: computed tomography
CAD: computer aided design
CAM: computer aided manufacturing

## Sources of funding

Not applicable.

## Ethical approval

Our report is not an experiment on human subjects.

The titanium plates used are already commercially available in Japan.

## Consent

Written informed consent was obtained from the patient or their parents for publication of this case report and accompanying images. A copy of the written consent is available for review by the Editor-in-Chief of this journal on request.

## Author contribution

Tadaaki Morotomi - Writing work, discussion and final approval; conception and design of the study.

Tomomi Iuchi - Literature review.

Mitsugu Fujita - Literature review.

Narihiko Hirano - Execution of the surgical step; acquisition of data.

Koji Niwa - Analysis and interpretation of imaging exams; analysis and interpretation of data.

## Registration of research studies

Our report is not ‘First in Human’ study, so we don't need to register.

The titanium plates used are already commercially available in Japan.

## Guarantor

Tadaaki Morotomi, M.D., Ph.D. will take the primary responsibility of the study.

## Provenance and peer review

Not commissioned, externally peer-reviewed.

## Declaration of competing interest

Not applicable.
